# Evaluation of Breastfeeding App Features: Content Analysis Study

**DOI:** 10.2196/37581

**Published:** 2022-10-26

**Authors:** Lauren M Dinour, Antoinette Pole

**Affiliations:** 1 Department of Nutrition and Food Studies College of Education and Human Services Montclair State University Montclair, NJ United States; 2 Department of Political Science and Law College of Humanities and Social Sciences Montclair State University Montclair, NJ United States

**Keywords:** breastfeeding, breastmilk expression, bottle feeding, infant food, infant health, infant care, consumer health informatics, mobile apps, smartphone, cross-sectional study

## Abstract

**Background:**

While a variety of health apps abound, less than half of adults in the United States report using a health app, despite the ubiquity of smartphones among users aged 18 to 49 years. Several studies have examined the use of breastfeeding apps; however, less is known about the types of features found on these apps and what factors might influence app ratings.

**Objective:**

This paper seeks to characterize breastfeeding apps, assess whether apps with higher user ratings differ from apps with lower user ratings in their tracking and nontracking features, and analyze whether the type and number of features predict user star ratings and whether an app is higher- or lower-rated.

**Methods:**

Using a cross-sectional design, a convenience sample of breastfeeding apps was culled from the Apple App Store (iOS) and Google Play Store (Android). Content analysis of the apps (N=82) was conducted using a schema of 87 items, which was then compiled into 9 topical indices for breastfeeding, bottle feeding, solid foods, infant health, infant care, technical characteristics, informatics, informational characteristics, and interactivity. Analysis consisted of descriptive statistics, the Mann-Whitney *U* test, and Spearman rank correlations. Linear regression and binary logistic regression analyses were conducted to determine which features predicted user star ratings.

**Results:**

On average, users rated breastfeeding apps 4.4 of 5 stars. Two-thirds of apps (n=54) were higher rated (≥4.5 stars), and one-third (n=28) were lower rated (<4.5 stars). Higher-rated apps offered more tracking features for breastfeeding, bottle feeding, solid foods, infant health, and infant care than lower-rated apps. The breastfeeding, solid-food, and technical indices explained 17% of user star ratings. For each additional breastfeeding and solid-food feature, we can expect to see a 27% and 35% increase, respectively, in user star ratings. Additionally, as the number of solid-food features increased, the odds that the app is higher rated increased 1.58 times.

**Conclusions:**

Our findings suggest user ratings are driven in part by tracking features, specifically those related to breastfeeding and solid foods. The proliferation of mobile health apps offers opportunities for parents and caregivers to track behaviors associated with infant feeding and other health metrics in a dynamic, detailed, and comprehensive manner. Hence, breastfeeding apps have the potential to promote and support breastfeeding among users.

## Introduction

Human milk is the gold standard for infant nutrition, and it is associated with improved maternal and infant health outcomes [1]. Many national and international health authorities recommend that infants be fed only human milk during the first 6 months of life, with continued breastfeeding alongside appropriate complementary foods for 1 year or longer [2-4]. Yet in the United States, only 1 in 4 infants born in 2018 were exclusively breastfed through 6 months, and about 1 in 3 were still breastfed at 12 months [5]. The reasons for early supplementation and breastfeeding cessation include inadequate knowledge; perceived inconvenience or embarrassment; medical conditions or lactation issues; lack of professional, family, and social support; early return to work; marketing of human milk substitutes; and societal norms and policies [6-8].

Mobile health (mHealth) technologies can address some barriers to breastfeeding by offering tracking features, data on user behavior, and information. The use of e-technologies has been associated with higher rates of breastfeeding initiation, exclusive breastfeeding at 4 weeks and 6 months, breastfeeding attitudes, and breastfeeding knowledge [9]. mHealth—the “medical and public health practice supported by mobile devices including mobile phones, patient monitoring devices, personal digital assistants...and other wireless devices” [10]—is on the rise due to the growth in smartphone ownership. In 2021, 85% of Americans owned a smartphone, up from just 35% in 2011. Rates are even higher among adults aged 18 to 29 years (96%) and 30 to 49 years (95%) [11].

Given the ubiquity of smartphone ownership, mHealth apps have become increasingly popular. By 2019, more than 45,000 [12] and 43,000 [13] mHealth apps were available in the Apple App Store and Google Play Store, respectively. An mHealth study by Krebs and Duncan [14] suggested individuals with more education, higher income, younger age, and Latino ethnicity were more likely to have downloaded a health app to track physical activity or dietary intake, help with weight loss, or learn exercises. Recent consumer data, however, show less than half of US adults have used or purchased health apps, and among individuals who report using a health app, more than half are upper or middle income [15].

The average childbearing age in the US is 26 years [16], which corresponds to a high rate of smartphone ownership. With limited formal structures for parental leave in the US, half of infants born in 2018 were breastfed for between 6 and 7 months. However, half of infants born in 2018 were exclusively breastfed for only 2 to 3 months [17]. Approximately one-third of infants receive human milk substitutes before 3 months of age [5]. Breastfeeding tends to be more heavily concentrated among certain racial and ethnic groups (ie, non-Hispanic Asian, non-Hispanic white, and Hispanic) and among college educated, higher income, and married women. Within the US, infants living in rural areas are less likely to have ever been breastfed than those living in urban areas, and infants living in the Southeast are less likely to be breastfed at 6 months than those living in other areas of the country [5].

In this nascent area of research, several studies have focused on one or more characteristics of infant-feeding smartphone apps. Mieso et al [18] performed a scoping review that addressed app development, user experience, and app effectiveness on breastfeeding outcomes. Studies of app development have reported the feasibility and need for smartphone apps to provide education, peer and professional support, and tracking features. User experience appears more positive than negative; apps were mostly helpful and reassuring, though some study participants noted apps were time-consuming, anxiety-provoking, burdensome, technically difficult, or provided questionable information. Only 3 studies examined app effectiveness, suggesting that apps are useful for capturing data and may help support exclusive breastfeeding and continuation of breastfeeding for 6 months [18].

Other studies have characterized the quality and content of infant-feeding smartphone apps available from the Apple App Store and Google Play Store. Cheng et al [19] evaluated 47 infant-feeding and activity apps in Australia, concluding the overall quality of information was poor, though apps were generally of moderate quality with regard to engagement, functionality, and aesthetics. Schindler-Ruwisch et al [20] similarly identified 50 breastfeeding apps in the US. The main interactive app features varied, and most apps only provided informational support (versus emotional, instrumental, or appraisal support). A plurality of apps included troubleshooting information related to breastfeeding and related issues, followed by information about breastfeeding in public [20]. Likewise, Sidhu et al [21] scored 41 US iPhone apps based on their features and content. Most apps (85%) offered features that assisted with promoting, tracking, or interpreting milk production. Among these, apps ranked in the top 200 in their respective categories within the Apple App Store received a significantly higher feature score compared to unranked apps. Finally, about one-third of apps in the sample contained educational content related to milk production; however, their content and diversity scores were low [21].

While previous scholarship has examined breastfeeding apps, little is known about the availability and comprehensiveness of features offered and their influence on user ratings. Because user ratings tend to drive downloads, these ratings potentially influence app adoption [22]. The aims of this study are to (1) provide descriptive statistics characterizing commercial breastfeeding apps in terms of their ratings, development, and other app details; (2) assess whether apps with higher and lower user star ratings differ in their tracking and nontracking features; and (3) determine whether the type and number of features predict user star ratings and whether an app is higher or lower rated.

## Methods

### Research Design

To best address the study aims, we chose a cross-sectional research design using content analysis. Given that apps are updated with new features over time, a longitudinal design was not appropriate. Our methods were informed by previous studies of infant-feeding apps [20,23] and other health apps [24-26]. This study was exempt from Institutional Review Board approval.

### Sample

**Figure 1:**
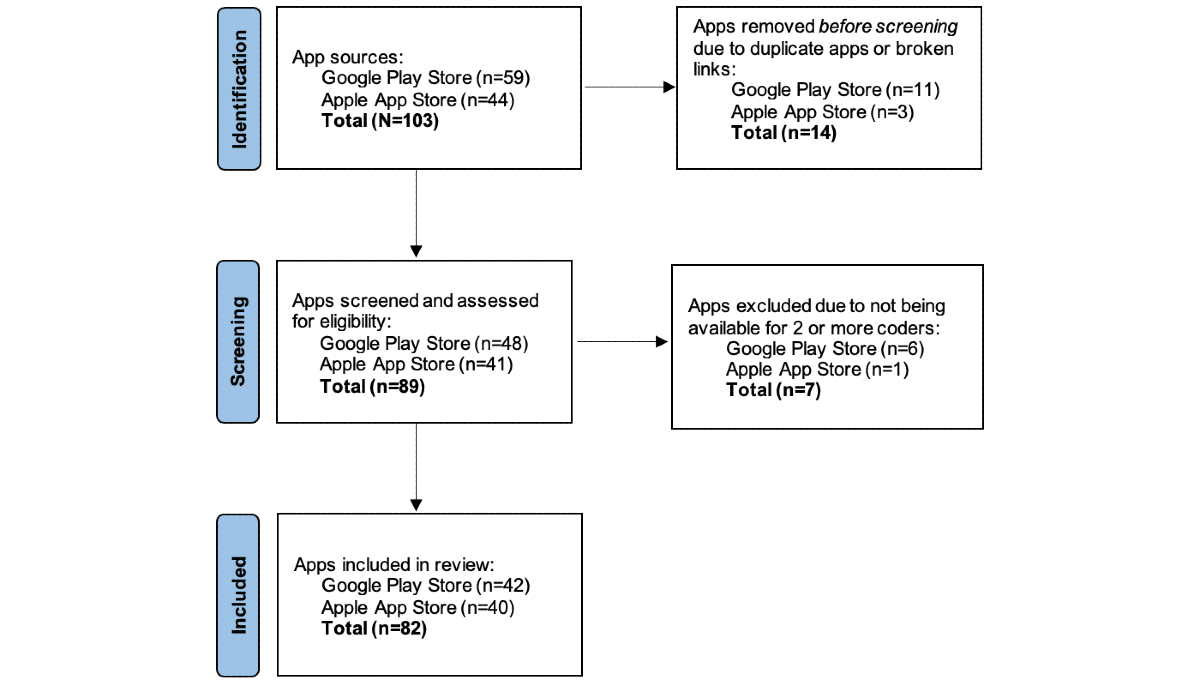
Smartphone breastfeeding app selection process.

To compile a convenience sample of breastfeeding apps, a graduate student in the US conducted a keyword search in the Apple App Store (iOS) and Google Play Store (Android) in fall 2018. A combination of keywords was used to search for English-language breastfeeding apps, including “breastfeeding” and “breastfeeding applications.” In January 2019, another graduate student created a sample in the same manner and cross-referenced it with the fall 2018 sample, increasing the sample size while also removing duplicates and dead links; the sample was finalized in February 2019. All relevant apps were included regardless of their cost. All apps were free except for 9; these 9 paid apps were downloaded for a combined cost of US $31.92 ($18.95 for 5 iPhone apps and $12.97 for 4 Android apps). A total of 40 iPhone and 42 Android apps were included in the final sample (N=82) of which 80 were free to users; only 2 paid iPhone apps remained in the final sample (Figure 1). The final sample is comparable to those of previous infant-feeding app studies, which included 41 to 77 apps [18,20,21].

### Measurement

To analyze the apps, a coding schema was created a priori based on existing studies of apps [20,24,27] and 2 breastfeeding textbooks [28,29]. The schema contained 87 distinct app characteristics and features. We defined features according to Sidhu et al [21] as any “opportunity for user interaction with the app (e.g., a button).”

Descriptive characteristics were derived from the app’s download page and included the name of the app, website link, download date, version number, date of last update, developer or seller name and affiliation (ie, commercial, government, nongovernment organization, university, unknown, or other), whether and which experts or end users were involved in the app development process, user rating (ie, number of stars out of 5), number of user reviews, app category (ie, medical, lifestyle, health and fitness, parenting, or other), language options, cost of basic and premium app versions, and age rating (not unlike a movie rating, each platform recommends the minimum maturity level of app content for end users by age, ie, >0, >4, >12, or >17).

Features were observed by navigating the downloaded app. Tracking features monitored breastfeeding, bottle feeding, solid foods, pumping and human milk expression, diapering, bathing, sleeping, infant growth and development, medication and vitamin use, vaccinations, temperature, illnesses, and well-child visits. Nontracking features included the ability to add notes, information, pictures, or videos; connect to a breast pump; print or export data; sync data with another program or device; use the app for more than one child or for multiple caregivers to use the app; customize features; receive static information (ie, articles, guidance, tips, checklists, product recommendations, frequently asked questions, pregnancy information, maps, graphs, or charts); share data with others (ie, other caregivers, health care providers, or social media); and interact with peers, lactation professionals, or others (Table 1).

**Table 1:** App features grouped by tracking and nontracking indices. Each variable was coded 0 (no), 1 (yes), or 2 (do not know). These summative indices only indicate the presence of a feature.

Indices		Features
**Tracking indices (range)**		
	Breastfeeding index (0-8)	Tracks start and stop times of a breastfeeding session Tracks time nursing per breast (left vs right) Tracks total time of a full breastfeeding session Tracks which breast (left vs right) was last nursed from Tracks number of pumping or milk-expression sessions Tracks amount of time per pumping or milk-expression session Tracks volume of milk per pumping or milk-expression session Tracks which breast (left vs right) was last pumped
	Bottle-feeding index (0-3)	Tracks number of bottle feeds Tracks time of bottle feeds Tracks volume of bottle feeds
	Solid-food index (0-4)	Tracks number of solid-food meals Tracks time of solid-food meals Tracks types of solid foods given Tracks amount of solid foods given
	Infant-health index (0-10)	Tracks infant’s weight over time Tracks infant’s length over time Tracks infant’s head circumference over time Compares infant’s growth to standards and averages Tracks milestones in physical development (eg, first tooth and first step) Tracks medication and vitamin use Tracks vaccines Tracks infant’s temperature Tracks infant’s illnesses Tracks infant’s well-child visits
	Infant-care index (0-7)	Tracks number of diaper changes Tracks time of diaper changes Tracks type of dirty diaper (urine vs feces) Tracks color of feces Tracks number of baths Tracks bath schedule Tracks nap and sleep schedule
**Nontracking indices (range)**		
	Technical index (0-15)	Ability to add notes to tracked data Ability to connect to breast pump Ability to add pictures or videos Ability to set notifications, alarms, or reminders Ability to print directly from app Ability to export data as email, text, pdf, spreadsheet, or eBook Ability to sync with cloud-based programs (eg, iCloud or Dropbox) Ability to copy data or sync to another device Ability to personalize app with infant’s picture, name, or date of birth Ability to use the app for more than one child at a time Ability for multiple caregivers to use the app and enter data Different themes for day and night Customizable features (eg, sound and content notifications) Audio content Video content
	Informatics index (0-3)	Provides maps or locations for where to feed or change an infant Provides graphs and charts Reports that support graphs and charts
	Informational index (0-5)	Provides articles, guidance, or tips Provides checklists Provides recommendations for products Provides frequently asked questions Provides pregnancy information
	Interactivity index (0-5)	Ability to share data with other caregivers or health care providers Ability to share with social media (eg, Facebook or Twitter) Peer support Ability to contact a lactation consultant or counselor Question and answer interface

### Data Collection

All apps were downloaded to a donated iPhone (iPhone 5S, iOS version 12.1.4; Apple Inc) or Android smartphone (Samsung Galaxy Note 2, Android version 4.4.2, or Samsung Galaxy S8, Android version 7.0; Samsung Electronics Co Ltd). Coders primarily used the shared iPhone and Android phones, aside from 1 student who used a personal iPhone to expedite coding. Coding of the apps began in March 2019 and was completed in December 2019. Two graduate students and 1 undergraduate honors student (with no prior involvement in the study) from different academic departments (including food and nutrition, law and governance, and political science and law) coded the apps.

Interrater reliability (IRR) was measured among the 3 coders with 3 possible outcomes. Agreement between all 3 coders was labeled as “complete agreement.” Agreement between 2 of 3 was considered “partial agreement.” When all 3 coders disagreed, we deemed this “no agreement.” In 7 apps, only 2 coders completed the coding; thus, the IRR was determined as “complete agreement” or “no agreement.” This might have occurred because one of the students used their personal phone or used a phone that was incompatible with a particular app version. The authors reviewed coder agreement on variables with partial or no agreement to determine the final coding decision. For continuous variables (eg, the number of languages, user ratings out of 5 stars, and number of user ratings) we used the most recent version of the app.

### Data Analysis

To address aim 1—characteristics of breastfeeding apps—descriptive statistics were used to characterize the sample and are reported as frequencies and percentages. Apps rated ≥4.5 stars were defined as higher-rated apps, while those rated <4.5 stars were considered lower-rated apps. Prior studies have used a cutoff of ≥4 stars [18,20]; however, the present sample had a skewed rating distribution, whereby only 16% (13 of 82) of apps were rated under 4 stars. Therefore, the 4.5-star cutoff was chosen to maximize variability in both groups. Nine summative indices were created by grouping like features by topic (Table 1). For aim 2—comparison of higher- and lower-rated apps—we used the Shapiro-Wilk test to assess the normality of the data and found that the indices were not normally distributed. We conducted Mann-Whitney *U* tests, which are appropriate for nonnormally distributed independent groups, to assess whether higher- and lower-rated apps differed by index.

To address aim 3—predictive relationships between user ratings and indices—we determined the Spearman rank correlation between the indices, user star ratings, and whether the app was higher rated or lower rated. All indices that were significantly correlated with the user star ratings were included in the linear regression model, except for bottle feeding, which was highly correlated (*r*=0.693) with the breastfeeding index. The same indices were entered into a binary logistic regression model to examine their ability to predict whether an app was higher or lower rated. Logistic regression results are reported as odds ratios (ORs) with 95% CIs. For all statistical tests, significance was defined at *P*<.05.

## Results

### Aim 1: Characteristics of Breastfeeding Apps

The sample was composed of 82 breastfeeding apps, including 40 iPhone and 42 Android apps (Table 2). On average, users rated breastfeeding apps 4.4 of 5 stars. Of the 82 apps reviewed, two-thirds (54) were higher rated and one-third (28) were lower rated. The number of user ratings per app ranged from 4 to 81,800.

**Table 2:** Descriptive characteristics of breastfeeding apps, overall and for apps with higher user star ratings and lower user star ratings.

Characteristics		Total (N=82), n (%)	Higher user star ratings^a^ (N=54), n (%)	Lower user star ratings^b^ (N=28), n (%)
**Platform**				
	iPhone	40 (49)	31 (57)	9 (32)
	Android	42 (51)	23 (43)	19 (68)
**Affiliations**				
	Commercial	61 (74)	37 (69)	24 (86)
	Nongovernmental organization	1 (1)	1 (2)	0 (0)
	Unknown	16 (20)	13 (24)	3 (11)
**Experts or end users involved in the development process**				
	Yes	21 (26)	16 (30)	5 (18)
	No	37 (45)	20 (37)	17 (61)
	Do not know	24 (29)	18 (33)	6 (21)
**Experts or end users involved**				
	Mothers	8 (10)	6 (11)	2 (7)
	Parents	6 (7)	5 (9)	1 (4)
	Neonatal intensive care unit staff	2 (2)	2 (4)	0 (0)
	Fathers	1 (1)	1 (2)	0 (0)
	Breast pump manufacturers	2 (2)	1 (2)	1 (4)
	Other	2 (2)	1 (2)	1 (4)
**Category**				
	Medical	36 (44)	27 (50)	9 (32)
	Lifestyle	3 (4)	1 (2)	2 (7)
	Health/fitness	15 (18)	4 (7)	11 (39)
	Parenting	26 (32)	21 (39)	5 (18)
	Productivity	1 (1)	1 (2)	0 (0)
	Tools	1 (1)	0 (0)	1 (4)
**Available languages**				
	English	82 (100)	54 (100)	28 (100)
	Spanish	18 (22)	17 (32)	1 (4)
	Chinese	13 (16)	13 (24)	0 (0)
**Cost of basic version**				
	US $0	80 (98)	53 (98)	27 (96)
	US $3.99	1 (1)	1 (2)	0 (0)
	US $4.99	1 (1)	0 (0)	1 (4)
**Age rating^c^ (minimum maturity level of end users)**				
	>0 years	41 (50)	23 (43)	18 (64)
	>4 years	31 (38)	24 (44)	7 (25)
	>12 years	8 (10)	6 (11)	2 (7)
	>17 years	2 (2)	1 (2)	1 (4)

^a^Apps with higher user star ratings are those with ≥4.5 stars.

^b^Apps with lower user star ratings are those with <4.5 stars.

^c^Age ratings differed by platform.

### Aim 2: Comparison of Higher- and Lower-Rated Apps

Mann-Whitney *U* tests were performed (Table 3) to determine differences between higher- and lower-rated apps. All indices were significant, and the mean ranks for all indices except the informatics, informational, and interactivity indices were greater among higher-rated apps than lower-rated apps. The breastfeeding and solid-food indices yielded the most notable differences in median scores between higher- and lower-rated apps.

**Table 3:** Mann-Whitney *U* test comparing apps with higher and lower user star ratings by index (N=82).

Indices			Higher user star ratings^a^ (n=54), median (range)		Lower user star ratings^b^ (n=28), median (range)		*U* statistic		*z* score		*P* value
**Tracking indices**											
	Breastfeeding index	7.0 (0-8)		4.0 (0-8)		483.500		–2.724		.006	
	Bottle-feeding index	3.0 (0-3)		3.0 (0-3)		592.000		–1.768		.001	
	Solid-food index	3.0 (0-4)		0.0 (0-3)		402.000		–3.699		.004	
	Infant-health index	3.5 (0-10)		4.0 (0-7)		579.500		–1.765		.004	
	Infant-care index	4.0 (0-7)		4.0 (0-7)		586.500		–1.712		.004	
**Nontracking indices**											
	Technical index	6.0 (0-12)		4.0 (1-10)		510.000		–2.418		.004	
	Informatics index	1.0 (0-1)		1.0 (0-2)		727.000		–0.326		.004	
	Informational index	0.0 (0-4)		0.5 (0-4)		611.000		–1.626		.004	
	Interactivity index	0.0 (0-4)		0.0 (0-2)		722.000		–0.494		.004	

^a^Apps with higher user star ratings are those with ≥4.5 stars.

^b^Apps with lower user star ratings are those with <4.5 stars.

### Aim 3: Predictive Relationships Between User Ratings and Indices

Table 4 illustrates the Spearman rank correlations between user star ratings, whether an app was higher versus lower rated, and the indices. The correlation between user star ratings and the solid-food index was positive and strong, while the correlations for breastfeeding, bottle-feeding, and technical indices were positive and moderate. The correlation between an app being higher rated and the solid-food index was positive and strong, while the correlations with the breastfeeding index were positive and moderate. Finally, the correlations between an app being higher rated and the bottle-feeding and technical indices were positive and weak.

A linear regression analysis was performed to determine whether breastfeeding, solid-food, and technical features predicted user star ratings (Table 5). The independent variables explained 17% of user star ratings (adjusted *R*^2^=0.172). The breastfeeding and solid-food indices were significant. For each additional breastfeeding feature, we can expect to see a 27% (*β*=.265, *P*=.047) increase in the user star rating, while each additional solid-food feature increases the user star rating by 35% (*β*=.354, *P*=.009).

A binary logistic regression analysis was performed to determine whether tracking features or nontracking features predicted higher user star ratings (Table 6). In the unadjusted bivariate analysis, there was a significant association between the breastfeeding, bottle-feeding, solid-food, and technical indices and the dependent variable. In addition, the odds of an app receiving a higher rating increased by 28% (OR 1.284, 95% CI 1.064-1.550) for each additional breastfeeding feature. Similarly, the unadjusted odds of an app receiving a higher rating increased by 68% for each additional bottle feeding (OR 1.683, 95% CI 1.112-2.548) and solid-food (OR 1.685, 95% CI 1.236-2.297) feature. The technical index also increased the odds that an app was higher rated. In the adjusted model, only the solid-food index remained significant. The odds of an app receiving a higher user star rating increased by 58% (OR 1.579, 95% CI 1.074-2.321) for each additional solid-food feature.

**Table 4:** Spearman rank correlations between user star ratings, higher versus lower user star ratings, and indices for the apps (N=82).

Indices		User star ratings (1-5), *ρ*	*P* value	Higher versus lower user star ratings^a^, *ρ*	*P* value
**Tracking indices**					
	Breastfeeding index	0.391	<.001	0.303	.006
	Bottle-feeding index	0.334	.002	0.242	.03
	Solid-food index	0.422	<.001	0.411	<.001
	Infant-health index	0.255	.02	0.196	.08
	Infant-care index	0.252	.02	0.190	.09
**Nontracking indices**					
	Technical index	0.343	.002	0.269	.02
	Informatics index	0.104	.35	–0.036	.75
	Informational index	–0.186	.09	–0.181	.10
	Interactivity index	–0.077	.49	–0.055	.62

^a^Apps with higher user star ratings are those with ≥4.5 stars; apps with lower user star ratings are those with <4.5 stars.

**Table 5:** Indices influencing user star ratings for the apps (N=82). Note: *R*=.451, *R*^2^=.203, adjusted *R*^2^=.172, and *F*_3_=6.625 (*P*<.001).

Variables	B	SE	*β*	*t* test^b^ (*df*)	*P* value	95% CI
Constant	4.025	0.136	N/A^a^	29.603 (78)	.001	3.754 to 4.295
Breastfeeding index	0.056	0.028	.265	2.023 (78)	.047	0.001 to 0.112
Solid-food index	0.109	0.041	.354	2.662 (78)	.009	0.027 to 0.190
Technical index	–0.025	0.027	–.136	–0.934 (78)	.35	–0.078 to 0.028

^a^N/A: not applicable.

^b^The *t* test was 2-tailed.

**Table 6:** Odds of indices predicting higher user star ratings for the apps (N=82). Note: Cox and Snell *R*^2^=.159, Nagelkerke *R*^2^=.219, and *χ*^2^_3_=14.168 (*P*=.003). The dependent variable was higher user star ratings (≥4.5 stars) set at 1, lower user star ratings (<4.5 stars) set at 0, and 1 set as the reference category.

Indices	Higher user star ratings			
	Unadjusted odds ratio (95% CI)	*P* value	Adjusted odds ratio (95% CI)	*P* value
Breastfeeding index	1.284 (1.064-1.550)	.009	1.142 (0.890-1.466)	.30
Bottle-feeding index	1.683 (1.112-2.548)	.01	N/A^a^	
Solid-food index	1.685 (1.236-2.297)	<.001	1.579 (1.074-2.321)	.02
Infant-health index	1.163 (0.997-1.357)	.06	N/A	
Infant-care index	1.218 (0.980-1.514)	.08	N/A	
Technical index	1.234 (1.041-1.463)	.02	0.977 (0.762-1.253)	.85
Informatics index	0.788 (0.337-1.846)	.58	N/A	
Informational index	0.724 (0.482-1.088)	.12	N/A	
Interactivity index	0.984 (0.516-1.877)	.96	N/A	
Constant	N/A		0.549	.30

^a^N/A: not applicable.

## Discussion

### Principal Findings

Our study builds on previous research of breastfeeding apps while expanding our understanding of what these apps offer by evaluating their features. Our sample is slightly larger than that of Mieso et al [18] and includes a greater percentage of free apps than earlier studies [18,20,21]. Similar to Schindler-Ruwisch et al [20], the sample draws upon a range of app categories, including medical, health and fitness, and parenting. Our cross-sectional review of apps occurred within a specified timeframe, akin to earlier studies [20,21].

Many characteristics of our sample reflect earlier studies of breastfeeding apps. For example, breastfeeding apps tend to be highly rated. Both Mieso et al [18] and Schindler-Ruwisch et al [20] found that nearly 70% of apps received user ratings >4 stars, and Mieso et al [18] showed that the average rating for breastfeeding apps was 4.3 of 5 stars. This is consistent with our findings. Similar to Mieso et al [18], the number of user reviews in our sample displayed a wide range.

Unsurprisingly, higher-rated apps offered more tracking features on all indices. In their qualitative analysis of maternal and infant health app user reviews, Biviji et al [30] found that across positive reviews, many users mentioned tracking features, including feeding, pumping, diapering, and sleep—akin to one-stop shopping. Conversely, there were complaints about apps with limited data-tracking abilities [30]. According to Mendiola et al [31], factors that predicted user ratings of health apps include usability, data export, and tracking. While the tracking component was negatively associated with user ratings, it was positively correlated with export and usability, both of which were positively associated with user ratings [31]. An alternative explanation as to why more features might appear in higher-rated apps is the release of new app versions that include new or updated features. Future studies should consider how tracking features correspond to other usability features and critically analyze the tracking features to determine their appropriateness to support infant-feeding goals. While informatics, informational, and interactivity features were not correlated with user star ratings, lower-rated apps had higher scores for these indices. Though few apps appeared to have these features, future studies might consider investigating their utility, since the study by Biviji et al [30] suggests that users desire these features.

In the regression models, the breastfeeding index predicted user star ratings; however, it did not predict whether an app was higher or lower rated. The former finding is to be expected, since breastfeeding tracking is the primary purpose of the apps. This is supported by Sidhu et al [21], who found that apps often had features that assisted with human milk tracking. However, we are unable to explain the latter finding, though it may be related to how we defined higher-rated apps.

Across both regression models, the solid-food index was significant. Solid food–tracking features allow users to continue with a familiar app that contains other tracking data (such as human milk or human milk–substitute consumption, diaper changing, or vaccinations) by carefully monitoring the introduction of new foods, which typically occurs on a weekly basis. This prolongs the usefulness of an app beyond a limited timeframe, again tapping into one-stop shopping [30]. Biviji et al [30] reported that positive app reviews emphasized tracking, highlighting feeding in particular. The authors demonstrated how users provided additional feedback on exporting data, additional tracking options, and data visualization, which might be incorporated into updated app versions [30]. Alternatively, the solid-food index might be a proxy for a feature not included in our study. Since our model only explains a small portion of the variance, we recommend an overall assessment of an app’s interface. For example, how seamless are the features? What are the advantages offered by one app over another? We also recommend a qualitative study of breastfeeding app users to gain greater insight into the reasons behind app adoption and features utilized.

### Limitations

While this study provides an overview of breastfeeding apps, there are several limitations. First, this was a convenience sample gathered between November 2018 and December 2019. We conducted a manual search with keywords, which may have resulted in missing some apps. Second, since new apps are frequently introduced to the market, this research only provides a snapshot in time; however, with a total of 82 apps, it still offers a comprehensive overview. Third, this research is limited to English-language apps in the US. Future studies should consider apps in other languages and in countries with higher rates of breastfeeding. Fourth, this study does not examine the apps’ clinical or scientific merits, but instead assesses features. Breastfeeding apps might contain content that is contrary to medical advice, and apps might not conform to national guidelines on infant feeding [19,23]; nevertheless, this was beyond the scope of this study.

### Conclusions

This study of breastfeeding apps demonstrates that user ratings are partially driven by tracking features, specifically those related to breastfeeding and solid foods. Nontracking features appear to be less important with regard to how users rate apps, though why this is the case remains unclear. Researchers should consider investigating this in the future. More importantly, the proliferation of mHealth offers opportunities for parents and caregivers to track behaviors associated with infant feeding and other health metrics in a dynamic, detailed, and comprehensive manner. In this way, breastfeeding apps have the potential to promote and support breastfeeding among users.

## Abbreviations

IRR: interrater reliability
mHealth: mobile health
OR: odds ratio
